# Like Cures Like: Pharmacological Activity of Anti-Inflammatory Lipopolysaccharides From Gut Microbiome

**DOI:** 10.3389/fphar.2020.00554

**Published:** 2020-04-30

**Authors:** Tzu-Lung Lin, Chin-Chung Shu, Young-Mao Chen, Jang-Jih Lu, Ting-Shu Wu, Wei-Fan Lai, Chi-Meng Tzeng, Hsin-Chih Lai, Chia-Chen Lu

**Affiliations:** ^1^Department of Medical Biotechnology and Laboratory Science, College of Medicine, Chang Gung University, Taoyuan, Taiwan; ^2^Microbiota Research Center and Emerging Viral Infections Research Center, Chang Gung University, Taoyuan, Taiwan; ^3^Department of Internal Medicine, National Taiwan University Hospital, Taipei, Taiwan; ^4^Bachelor Degree Program in Marine Biotechnology, College of Life Sciences, National Taiwan Ocean University, Keelung, Taiwan; ^5^Department of Laboratory Medicine, Linkou Chang Gung Memorial Hospital, Taoyuan, Taiwan; ^6^Division of Infectious Diseases, Department of Internal Medicine, Linkou Chang Gung Memorial Hospital, Taoyuan, Taiwan; ^7^Department of Medicine, College of Medicine, Chang Gung University, Taoyuan, Taiwan; ^8^School of Pharmaceutical Sciences, Xiamen University, Xiamen, China; ^9^Central Research Laboratory, Xiamen Chang Gung Allergology Consortium, Xiamen Chang Gung Hospital, Xiamen, China; ^10^Research Center for Chinese Herbal Medicine and Research Center for Food and Cosmetic Safety, College of Human Ecology, Chang Gung University of Science and Technology, Taoyuan, Taiwan; ^11^Department of Chest Medicine, Internal Medicine, Fu Jen Catholic University Hospital, Fu Jen Catholic University, New Taipei City, Taiwan; ^12^Department of Respiratory Therapy, Fu Jen Catholic University, New Taipei City, Taiwan

**Keywords:** lipopolysaccharides, microbiota, proteobacteria, bacteroidetes, TLR4, immune modulation

## Abstract

Gut microbiome maintains local gut integrity and systemic host homeostasis, where optimal control of intestinal lipopolysaccharides (LPS) activity may play an important role. LPS mainly produced from gut microbiota are a group of lipid-polysaccharide chemical complexes existing in the outer membrane of Gram-negative bacteria. Traditionally, LPS mostly produced from Proteobacteria are well known for their ability in inducing strong inflammatory responses (proinflammatory LPS, abbreviated as P-LPS), leading to septic shock or even death in animals and humans. Although the basic structures and chemical properties of P-LPS derived from different bacterial species generally show similarity, subtle and differential immune activation activities are observed. On the other hand, frequently ignored, a group of LPS molecules mainly produced by certain microbiota bacteria such as Bacteroidetes show blunt or even antagonistic activity in initiating pro-inflammatory responses (anti-inflammatory LPS, abbreviated as A-LPS). In this review, besides the immune activation properties of P-LPS, we also focus on the description of anti-inflammatory effects of A-LPS, and their potential antagonistic mechanism. We address the possibility of using native or engineered A-LPS for immune modulation in prevention or even treatment of P-LPS induced chronic inflammation related diseases. Understanding the exquisite interactive relationship between structure-activity correlation of P- and A-LPS not only contributes to molecular understanding of immunomodulation and homeostasis, but also re-animates the development of novel LPS-based pharmacological strategy for prevention and therapy of chronic inflammation related diseases.

## Introduction

### Lipopolysaccharides in General

**Figure 1:**
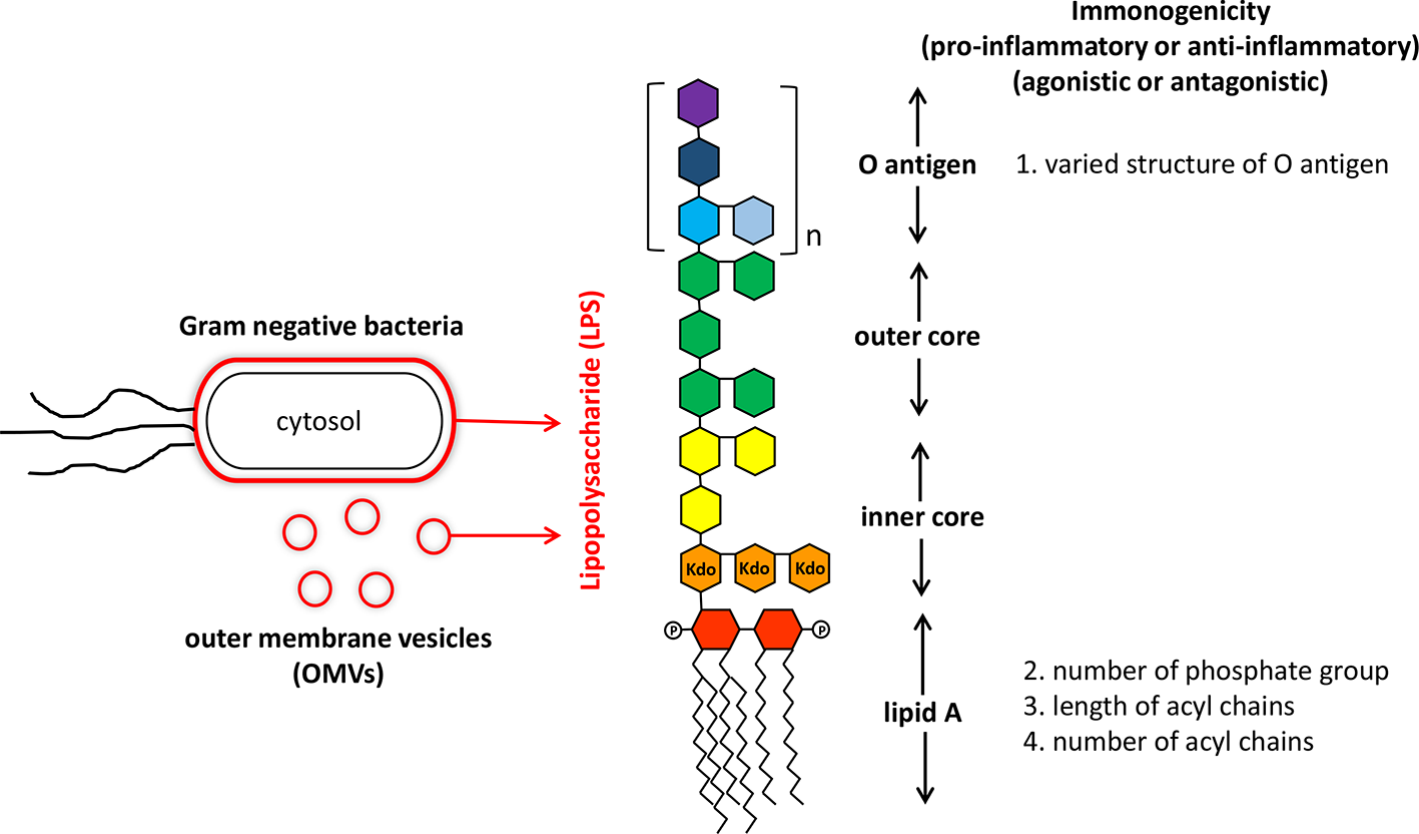
Structure and immunogenicity of lipopolysaccharide from Gram-negative bacteria. Lipopolysaccharide (LPS) is the major component of outer membrane of Gram-negative bacteria and also is anchored on outer membrane vesicles (OMVs) secreting by live Gram-negative bacteria. The structure of LPS consisted of a core lipid structure (core polysaccharide and lipid A) and polysaccharide components (O antigen). The immunogenicity of LPS is affected by variations in the O antigen structure (sugar composition, length, and permutation), and modification of lipid A (number of phosphate group, number, and length of acryl chains).

Lipopolysaccharides (LPS) mainly derived from gut microbiome are chemical molecules located in the outer membrane of Gram-negative bacteria (Sperandeo et al., 2017). It is a pathogen associated molecular pattern (PAMP) molecule consisted of a core lipid structure and polysaccharide components (Nativel et al., 2017). Traditionally, LPS are best known for their eliciting strong immune responses in a variety of eukaryotic species ranging from insects to animals and humans (Alexander and Rietschel, 2001). Increased concentration of LPS in the sera is also closely related to development of sepsis and even mortality (Fink, 2014). Detailed structural analysis indicated that LPS consist of three parts: lipid A, a core oligosaccharide and an O antigen of oligo- or polysaccharide chain (Alexander and Rietschel, 2001). The conserved lipid A entity buried within the Gram-negative bacterial outer membrane is basically composed of a *β*(1 → 6)-linked glucosamine disaccharide backbone that is phosphorylated at positions 1 and 4 of the disaccharide, and acylated at positions 2 and 3 of each monosaccharide (Steimle et al., 2016). Covalently attached to lipid A is the core oligosaccharides that link to lipid A through 3-deoxy-d-*manno*-oct-ulosonic acid (Kdo), and show variance among LPS purified from different bacterial species (Amor et al., 2000). Located on the outermost part of LPS, the highly versatile O-antigen sugar chain is characterized by significant variation of sugar length, composition, arrangement and the linkages between monosaccharides in different bacterial species, different bacterial strains of the same species, or even different bacterial clones of the same bacterial strain (intrastrain LPS heterogeneity) (Lerouge and Vanderleyden, 2002) (**Figure 1**).

### Proinflammatory Lipopolysaccharides

Traditional proinflammatory LPS (P-LPS) are mostly derived from the phylum Proteobacteria such as those from *Escherichia coli*. They have been established to play important roles in increasing the oxidative stress and over-production of inflammatory cytokines/chemokines. Functional activities of P-LPS are widely identified in not only mammals (Wiese et al., 1999), but also other animals including chicken and fish (Komori et al., 2015). While the three parts of P-LPS may all patriciate in modulation of immune activities, the lipid A is the primary immunostimulatory moiety of P-LPS. After lysis of bacterial cells, exposed lipid A binds to cell surface receptors of target cells such as macrophages and dendritic cells (DCs), initiating downstream inflammatory signaling components. Subsequently, fever, inflammation, and even septic shock are induced. Release of bacterial LPS can not only be achieved after bacterial lysis, but also through secreting outer membrane vesicles (OMVs) by live Gram-negative bacteria (Vanaja et al., 2016) (**Figure 1**). Though proinflammatory, the biological activity of P-LPS molecules show a range of differential activities on target cells. Such effects depend on various factors, including bacterial origin and P-LPS structure, composition, and concentrations (Chantratita et al., 2013). Although lipid A moiety may be the most active component of LPS, the O-antigen part also participates in immune regulation activity (Kadowaki et al., 2013) (**Figure 1**).

### Cascades of Proinflammatory Lipopolysaccharides Activated Inflammations

**Figure 2:**
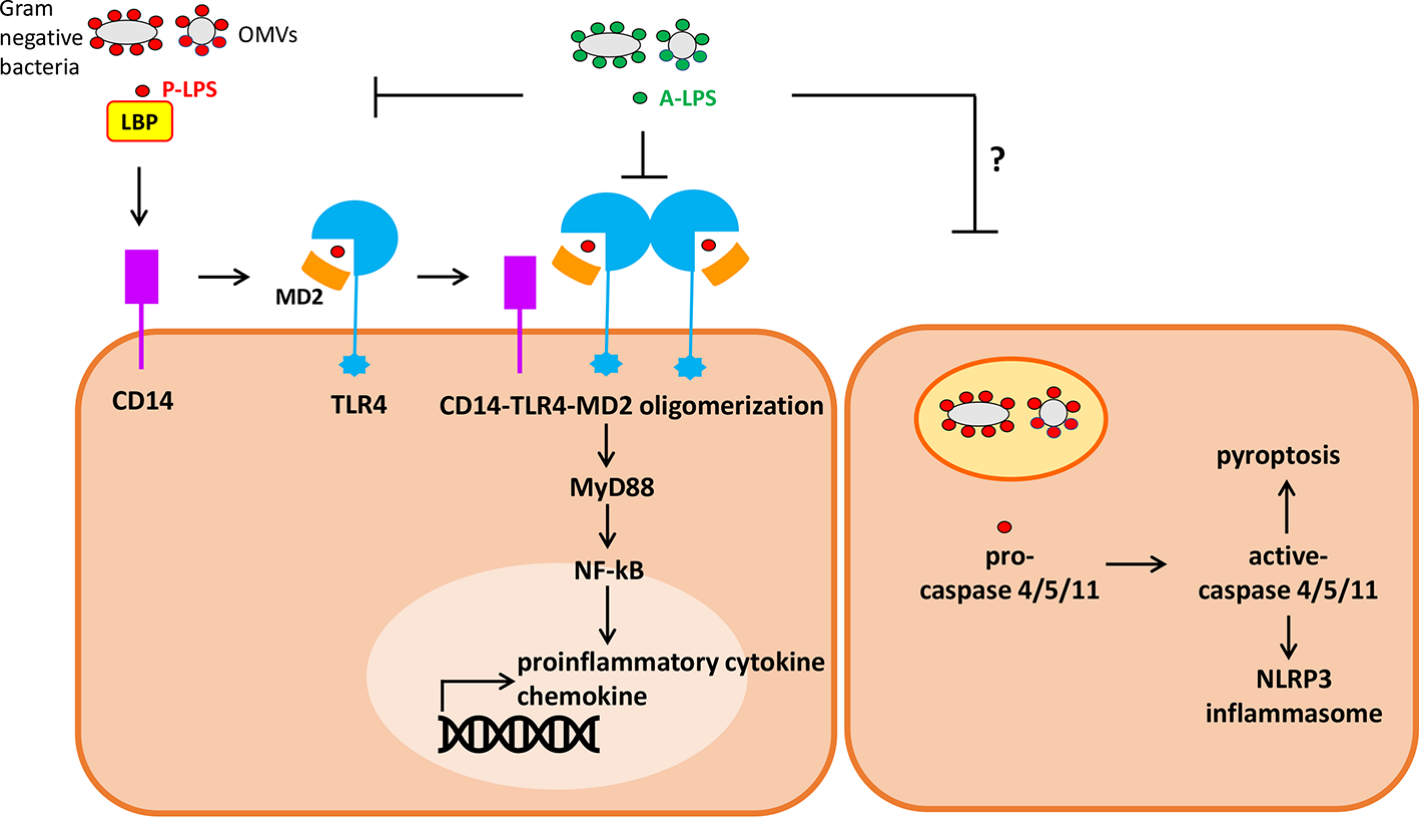
Simplified diagram of extracellular and intracellular signaling by P-lipopolysaccharides (LPS) (pro-inflammatory) and A-LPS (anti-inflammatory) on host cells. (left) P-LPS is bound by LPS binding protein (LBP), passed to CD14, then transferred to MD-2 and TLR4. P-LPS-induced CD14-TLR4-MD2 receptor oligomerization promotes activation of the transcription factor NF-κB through MyD88-dependent signaling cascade, then induces the expression of genes encoding proinflammatory cytokines and chemokines. A-LPS may antagonize P-LPS-induced activation of TLR4 through interfering the interactions between P-LPS and LBP as well as P-LPS and TLR4-MD2. (Right) P-LPS can be transported into cytosol from endosome containing Gram-negative bacteria or outer membrane vesicles (OMVs). In cytosol, LPS can be sensed and then activates caspase-4/5/11. This may lead to activation of NLRP3 inflammasome and pyroptosis of the cells.

P-LPS act as important molecules initiating local and even systemic inflammations in the host. While there are multiple TLR4-independent P-LPS sensing pathways, TLR4 is currently regarded as a major cell surface pattern recognition molecule receptor (PRR) responsible for initiation and sustaining the inflammatory responses in the host (Nativel et al., 2017; Mazgaeen and Gurung, 2020). Upstream interaction between P-LPS and cell surface TLR4 ignites the whole cascade of downstream signaling (**Figure 2**). P-LPS does not directly bind to TLR4. In body fluids, P-LPS micelles first interact with LPS-binding protein (LBP), an acute-phase protein, forming P-LPS/LBP micelles. Sequentially, P-LPS/LBP micelles then interact with CD14. Afterwards, the resulting complex interacts with TLR4/MD-2, leading to oligomerization of TLR4/MD-2/CD14 complex (Kim and Kim, 2017). Subsequently, aggregated LPS-TLR4 complex (P-LPS/TLR4/MD-2/CD14) activates cells through eliciting the NF-κB signaling pathway, leading to increased production and secretion of abundant pro-inflammatory cytokines such as IFN-γ, TNF-α, interleukin (IL)-1β and interleukin (IL)-8 (but little or no IL-4, IL-13 or IL-5), and chemokines, such as monocyte chemoattractant protein 1 (MCP-1) (Chow et al., 1999; Alexander and Rietschel, 2001). Alternatively, P-LPS may also activate immune cells by TLR4 independent pathway. Recent studies indicated that LPS can be transported into cytosol by OMVs and guanylate-binding proteins. In cytosol, LPS can be sensed by the noncanonical inflammasome and directly binds to its intracellular receptor caspase-4/5/11. This may lead to activation of NLRP3 inflammasome and pyroptosis of the cells. The LPS-triggered cytosolic activation of noncanonical inflammasome may play important roles in development of sepsis (Pfalzgraff and Weindl, 2019; Mazgaeen and Gurung, 2020).

The P-LPS induced lung inflammations are described here, where sequential multiple biochemical steps occur, leading to final lung pathogenesis development. Following P-LPS-TLR4 interaction, TLR4/MyD88 signaling pathways (Lu et al., 2008) in alveolar macrophages are induced, leading to increased release of proinflammatory cytokines/chemokines such as IL-1β, IL-6, IL-8, and MCP-1 (Atamas et al., 2013). These subsequently provoke infiltration of various over-activated immune cells including macrophages, neutrophils, and CD8+ T and B lymphocytes (Asti et al., 2000). Proteolytic enzymes activities such as those from elastase, matrix metalloproteinase (MMP-2, MMP-9, MMP-12) and cathepsins are also increased in lung (Davey et al., 2011). This leads to enhanced degradation of collagen and matrix proteins which further enhance inflammation. Together with other exogenous and endogenous oxidative stresses, the lung parenchyma starts to be damaged. Cell injuries further activate proteases and generate danger-associated molecular patterns (DAMPs) such as hsp70 (Hulina-Tomaskovic et al., 2019) or high mobility group box 1 (HMGB-1) protein (Gangemi et al., 2015). DAMPs continue to activate inflammasome in macrophage, leading to reduced mitochondrial function, cell senescence, apoptosis, and necrosis (Faner et al., 2012).

### Proinflammatory Lipopolysaccharides–TLR4 Interaction in Modulation of Immunity and Inflammation

P-LPS derived from the gut microbiota of animals and humans have been shown to cause or contribute to development of chronic inflammation related diseases. The P-LPS levels in blood plasma are normally low, but are found to be elevated in many chronic inflammation related diseases. These include infections and sepsis (Opal, 2010), obesity and type 2 diabetes (Hersoug et al., 2016), gum disease (Brown, 2019), chronic obstructive pulmonary disease (COPD) (Aul et al., 2012; Kobayashi et al., 2013; Gupta et al., 2015), non-alcoholic fatty liver disease (NAFLD) (Fuke et al., 2019), colitis associated cancers (Waldner and Neurath, 2015), and even neurodegenerative diseases such as Alzheimer’s disease, Parkinson’s disease, and amyotrophic lateral sclerosis (Brown, 2019). Concordantly, dysbiosis in the gut microbiome may increase the production and release of microbial P-LPS which activate gut inflammatory response. Pro-inflammatory cytokines produced in gut can systematically affect host physiology, such as stimulating the afferent vagal nerve which in turn impacts the hypothalamic-pituitary-adrenal (HPA) axis and induces symptoms associated with depression (Simkin, 2019). Gathering evidences strongly indicate the causative relationship between increased P-LPS concentration in body fluids and development of chronic inflammations related diseases. Therefore, it is possible that these diseases may be ameliorated by either decreasing P-LPS levels or antagonizing P-LPS-induced inflammations (Brown, 2019).

P-LPS do not just provoke unfavorable inflammations. These molecules may also contribute to restoring immunity-compromised diseases due to gut microbiota dysbiosis. Using an animal model of antibiotics-induced dysbiosis and bacterial lung infection, previous work has shown that oral supplementation with commensal flora derived P-LPS improves lung defense against *E. coli*-induced pneumonia. Thus, P-LPS derived from the gut microbiota may increase host lung immunity in this context (Chen, Chen et al., 2011). The mechanism underlying the effects of LPS modulatory effects remains to be further examined.

Efficacy of P-LPS activation activity can be significantly affected by variations in the sugar composition, length and permutation, and modification of lipid A, core, and/or O-antigen structure (Fedele et al., 2008). On the other hand, expression levels or structural variations in the target LBP, CD14, and TLR4 protein complex of host cells may also affect the immune-stimulation activities (Hajjar et al., 2017). In brief, TLR4-MD-2 receptor complex recognizes variations in P-LPS molecule of a particular Gram-negative bacterium and uses multiple sites in interaction. In this situation, subtle tuning of the earliest interaction between the host cell surface and pathogen P-LPS occurs, which affects P-LPS affinity and the subsequent activation activity (Maeshima et al., 2015). For example, LPS derived from Chlamydiaceae shows reduced binding affinity for LBP and CD14, and exhibits weak biological activity against host immune cells such as monocytes (Tsutsumi-Ishii et al., 2008). Besides, studies of LPS derived from *Bordetella pertussis* indicate that distinct charged and uncharged amino acids in TLR4 and MD-2 proteins determine the binding affinity between lipid A and TLR4/MD-2, affecting subsequent macrophage activation (Maeshima et al., 2015). Another example is the involvement of LBP and soluble CD14 (sCD14) in modulation of LPS response in a concentration dependent manner. While low concentration of LBP enhances P-LPS responses, high LBP concentration during acute inflammation and infection inhibits P-LPS bioactivity in contrast. On the other hand, in body fluid, systematic sCD14 may distract P-LPS from membrane-bound CD14 (mCD14) and inhibit TLR4 signaling. Dual stimulatory and inhibitory mechanisms of LPB and sCD14 may therefore exist to modulate the inflammations in infected host (Kitchens and Thompson, 2005). Subtle interactive variations at the host-pathogen interface thus fine-tune the host immune responses to specific P-LPS.

### Microbiota Anti-Inflammatory Lipopolysaccharides Counteract Proinflammatory Lipopolysaccharides to Achieve Homeostasis

LPS are mostly produced from gut microbiota, besides those from external infections, the respiratory tract and gum (Brown, 2019). Gut microbiota are mostly strict anaerobes (97%), and play multiple important roles in maintaining host intestinal homeostasis and promoting health (Alexandre et al., 2018). Basic composition of the human gut microbiota is composed of bacteria from the phyla Firmicutes (64%, mainly Gram-positive *Clostridium*, *Bacillus*, *Lactobacillus*, and *Enterococcus* species), and *Bacteroidetes* (23%, mostly Gram-negative *Bacteroides* and *Prevotella* species). Other phyla include Proteobacteria (1–8%), Actinobacteria (3%), and low numbers of the phyla *Fusobacteria*, Verrucomicrobia, and TM7 (2%). By contrast, fungi and archaea comprise less than 1% of the total gut microbiota (Cardinelli et al., 2015). Under normobiosis situation, hosts do not develop gut inflammatory phenotypes. This may be due to a harmonic habituating relationship of microbiota bacteria and optimal control of P-LPS activity in the intestine, leading to host intestinal homeostasis and beyond. Within the gut microbiota, intestinal Proteobacteria work as major contributors of P-LPS synthesized. Among these, an average of 14% of total P-LPS produced is of *E. coli* origin in healthy people, and 5.2% in human microbiome project 1(HMP1) donors (Alexandre et al., 2018).

Recent studies indicated that total LPS prepared from the consortium of gut-resident microbes potently antagonize the *E. coli* LPS-host TLR4 signaling pathway (Alexander and Rietschel, 2001; d’Hennezel et al., 2017). Further metagenomic sequencing delineated the strain level contributions to the gut LPS pool and found that bacteria across the members of the order Bacteroidales produce antagonistic forms of LPS (A-LPS), thus driving immune silencing for the entire microbial community. Especially, Bacteroidetes species may contribute up to 79% of the A-LPS produced in healthy people and 92.4% of that in HMP1 samples. The estimated average ratio of Bacteroidetes-to-*E. coli* LPS in the gut would be between 6:1 in healthy people and 18:1 in the HMP1 cohort (Alexandre et al., 2018). The abundances of the *Bacteroides* species, and therefore their likely contribution to the whole LPS pool are started to be unraveled to play an important role in intestinal immune homeostasis.

### Anti-Inflammatory Lipopolysaccharides Is Characterized by Structure Variations of Lipid A

It is established that natural heterogeneity observed in the lipid A structure portion of LPS may produce differential modulatory effects on immune responses (Chilton et al., 2013). The underlying silencing mechanism of A-LPS is subsequently deciphered to be closely related to their status of lipid A acylation in contrast to P-LPS, where hypoacylation is frequently observed (Coats et al., 2007; d’Hennezel et al., 2017). For example, tetra- or penta-acylation of A-LPS in contrast to the well-established hexaacyl-lipid A of *E. coli* (Reife et al., 2006) is identified. Such hypoacylation characteristic of lipid A structure is expected to be found in many Bacteroidetes bacteria including *Bacteroides dorei*, *Bacteroides fragilis*, *Bacteroides ovatus*, *Bacteroides thetaiotaomicron*, and *Bacteroides uniformis …* etc. (Poxton and Edmond, 1995; d’Hennezel et al., 2017; Jacobson et al., 2018). Another example is the hypoacylated LPS from a foodborne pathogen *Campylobacter jejuni* that only moderately induces TLR4 dependent immune response (Korneev et al., 2018). Furthermore, *Shigella flexneri* 2a that contains a mixture of hexaacylated, pentaacylated, and predominantly tetraacylated lipid A in its LPS also significantly decreases its stimulatory effects on NF-kappaB signaling pathway in contrast to the hexaacylated *E. coli* LPS (Rallabhandi et al., 2008). On the other hand, structurally similar, pentaacylated LPS of *Porphyromonas gingivalis* and *B. thetaiotaomicron* initiate significantly different innate immune responses (Berezow et al., 2009), highlighting the importance of exquisite structural variations of lipid A of LPS in immune regulation.

Besides hypoacylation, hypophosphorylation of the diglucosamine backbone also decreases LPS toxicity. The A-LPS derived from the intestinal mucosa-associated bacteria *B. thetaiotaomicron* and *Prevotella intermedia* contain monophosphorylated lipid A (MPLA). These A-LPS showed moderate immune-stimulating functions and could work as immunological adjuvants for antigen-specific immune responses (Chilton et al., 2013). As the immune silencing effect *via* A-LPS seems to be an intrinsic characteristic in healthy hosts, the current belief that robust activation of TLR4 signaling by gut microbiome derived LPS is therefore to be carefully reconsidered (d’Hennezel et al., 2017).

### Anti-Inflammatory Lipopolysaccharides Combats Inflammation Induced by Proinflammatory Lipopolysaccharides

A-LPS may show antagonistic effects on P-LPS activity. Taking modulation of colitis by use of A-LPS as an example, mice harboring low levels of Enterobacteriaceae (main P-LPS producer) and high Bacteroidetes (main A-LPS producer) showed intestinal low endotoxicity and maintained mucosal immune homeostasis. By contrast, mice harboring a highly endotoxic gut microbiota (with high Enterobacteriaceae and low Bacteroidetes levels) were prone to develop colitis. In concordance, administration of *E. coli* JM83 (wild-type P-LPS) to mice exacerbated colitis, whereas a mixture of *E. coli* JM83 and *E. coli* htrBPG (mutated LPS, with a lower endotoxicity similar to that of Bacteroidetes) prevented colitis development in mice. These results indicated that the A-LPS produced by the intestinal microbiota may counteract P-LPS dominantly induced colitis development in mice (Gronbach et al., 2014). Another example was *B. dorei* that produces an antagonistic A-LPS affecting the susceptibility of children to allergies and autoimmunity (Vatanen et al., 2016). Besides, *B. fragilis* and *B. ovatus* also alleviated the P-LPS-induced inflammation in mice (Tan et al., 2019), and *Bacteroides vulgatus* and *B. dorei* ameliorated endotoxemia, decreased gut microbial LPS production, and suppressed proinflammatory immune responses (Yoshida et al., 2018). Recently, the intestinal inflammation-reducing properties of weak agonistic A-LPS derived from *B. vulgatus* were reported to be due to inducing a special type of endotoxin tolerance, mainly through the MD-2/TLR4 receptor complex axis in CD11c^+^ cells of intestinal lamina propria (Steimle et al., 2019).

On top of these Bacteroidale bacteria, some Proteobacteria bacteria such as *Rhodobacter capsulatus* and *Rhodobacter sphaeroides* also owned A-LPS that lack potent agonist activity (Alexander and Rietschel, 2001). What’s more, A-LPS derived from Rhodobacter, such as E5531 (Rc-LPS derived) and E5564 (eritoran tetrasodium, developed from E5531) further showed potent antagonism of P-LPS-mediated cellular activation (Christ et al., 1995). E5564 was subsequently shown to prevent chronic airway hyperreactivity and inflammation to inhaled P-LPS (Savov et al., 2005). Besides, not only *in vitro* effects, A-LPS also showed protective effect on mice suffered from P-LPS-induced lethality (Barochia et al., 2011). Another example is that, in contrast to P-LPS prepared from *E. coli*, A-LPS prepared from two bacterial strains, the commensal *Endozoicomonas* sp. and the opportunistic bacteria *Pseudoalteromonas* spp., that are associated with the sponge *Suberites domuncula* are non-toxic for mammals. The relatively low acylation of the lipid A of *Pseudoalteromonas* sp. 1A1 and *Endozoicomonas* sp. HEX311 may be a possible reason to explain their A-LPS characteristics (Garderes et al., 2015).

Results from our study also indicated that lipid A of A-LPS derived from the bacterium *Parabacteroides goldsteinii* MTS01 is expected to be pentaacylated, based on genome comparison and structural determination results (unpublished). *P. goldsteinii* MTS01 derived A-LPS (Pg-LPS) antagonizes *E. coli* O111:B4 LPS induced TNF-α production in macrophages RAW264.7, IL-1β in human PBMC, and over-activation of BCR signaling in B cells (Xu et al., 2008) and reverses the expression of CS-induced inflammations-related genes, leading to alleviating the pathogenesis of COPD in lung and colon tissues (unpublished). Pg-LPS seems to be consistent in their immune inhibiting effect against many different kinds of cells. On the other hand, the antagonistic potential of hypoacylated LPS from *R. sphaeroides* (Rs-LPS) is not consistently seen against many immune cells, and is dependent on the cell sources of mammalian species (Doring et al., 2017). Therefore, complicated and minute interactions occur between LPS and immune cells, which has to be taken into consideration for subsequent development of clinical applications.

### Anti-Inflammatory Lipopolysaccharides Competes With Proinflammatory Lipopolysaccharides in TLR4 Signaling Pathway

“Competition” may be the underlying mechanism that hypoacylated A-LPS antagonizes P-LPS activity (**Figure 2**). Previous study has shown that tetra-acylated LPS derived from *P. gingivalis*, and penta-acylated msbB LPS derived from an *Escherichia coli* mutant strain antagonized the capability of hexa-acylated *E. coli* LPS to activate the TLR4 signaling complex in human endothelial cells (Coats et al., 2005). While expression levels of TLR4 do matter in modulating the efficacy of LPS-dependent antagonism, MD-2 seemed to act as the principal molecular component responsible for the antagonistic effects, due to fact that msbB and *P. gingivalis* LPS could directly bind to hMD-2 (Coats et al., 2005). Subsequent studies showed that these antagonistic A-LPS might utilize at least two distinct mechanisms to block *E. coli* P-LPS-dependent activation of hTLR4: i) directly compete with the *E. coli* P-LPS for the same binding site on hMD-2, and ii) to inhibit complexes interaction between *E. coli* P-LPS-hMD-2 and hTLR4. Besides hMD-2, hTLR4 also participated in species-specific recognition of msbB and *P. gingivalis* A-LPS at the hTLR4 complex (Coats et al., 2007). Results obtained from crystal protein structures studies, as well as targeted mutagenesis analyses of the receptor complex might give some more hints. Combination of intricate electrostatic and hydrophobic interactions primarily occurring within the MD-2 co-receptor, with a contribution from TLR4, may contribute to the species-specific recognition of lipid A by host cells (Oblak and Jerala, 2015). As underacylated LPS has emerged as a novel mechanism utilized by microbiota bacteria to optimally modulate host innate immune responses, these LPS may be developed as prime therapeutic candidates for neutralizing Gram-negative bacteria initiated bacterial sepsis.

### Lipopolysaccharides and TLR4 as Targets of Immune Modulation

Varying LPS structures may be used as a means by which Gram-negative bacteria control host immune status. Therefore, engineering LPS structure and chemical properties can be used as a strategy for development of novel immune-regulation agents. For example, controlling the acylation and phosphorylation status of lipid A may be considered. The position of phosphate group on lipid A may change the potency of LPS (Coats et al., 2009; Coats et al., 2011). On the other hand, lipid A without phosphate group due to alkaline phosphatase activity produced from host can even lead to formation of the non-immunostimulatory LPS, as illustrated by the *Eurypmna scolopes*-*Vibrio fischeri* symbiotic association (Rader et al., 2012). Another example is the lipid A of P-LPS of *V. fischeri* in which heterogeneous mixtures of mono- and diphosphorylated disaccharides, with variable acylation situations from tetra- to octaacylated were identified. These uncommon phosphoglycerol entity and carbohydrate compositions of lipid A may modulate the close interaction between *V. fischeri* and *E. scolopes* during symbiotic development (Phillips et al., 2011; Post et al., 2012), where imbalanced bacteria-host immune response is prevented.

Another example of application by lipid A modification is the mono-phosphorylated lipid A species (MPL) which is less toxic compared to conventional lipid A, and is the first vaccine adjuvant approved by the Food and Drug Administration (FDA) in more than 70 years (Needham et al., 2013). In contrast to the wild-type *E. coli* LPS which mainly acts on TLR4-MyD88 signaling, MPL preferentially effects through TRIF which is less inflammatory (Needham et al., 2013). On top of these, addition of aminoarabinose residues onto lipid A of *Burkholderia cenocepacia* P-LPS enhanced binding efficacy of lipid A to TLR4-MD-2 complex. This might initiate strong activation, despite the lipid A moiety having only five acyl chains (Di Lorenzo et al., 2015). The engineering of lipid A of P-LPS in a human pathogen *B. pertussis* for better control of P-LPS-TLR4 activity is also described. Glucosamine is added into lipid A moiety, leading to promote TLR4 activation in human macrophages. In parallel, site-directed mutagenesis together with a NF-κB reporter assay are also used to screen TLR4 and MD-2 mutants with changed amino acid residues that change species-specific responses. Results indicate that some uncharged amino acids in both TLR4 and MD-2 are involved in recognition of penta-acylated *B. pertussis* lipid A (Maeshima et al., 2015). These amino acids may be considered as research targets for optimizing P-LPS-TLR4 interaction and activation activity.

Besides lipid A, engineering of O-antigen may also be taken into consideration. Oral or intradermal administration of LPS derived from the bacterium *Pantoea agglomerans*, a bacterium that grows symbiotically in wheat, produce prophylactic and anti-tumor effects, even though no serious side-effects are identified. The main LPS structural difference that involves such biological effects lies in the structure of the O-antigen polysaccharides (Nishizawa et al., 1992). On the other hand, O-antigen defective LPS derived from certain *E*. *coli* strains may be anti-inflammatory. For example, the probiotic *E. coli* strain Nissle 1917 given orally to mice exert local and systemic anti-inflammatory effects in a model of LPS-induced sepsis (Arribas et al., 2009). Further analysis show that this strain has a defective LPS biosynthesis pathway that results in production of truncated variable oligosaccharide-antigen chains and gives the bacteria a semi-rough phenotype. Such characteristic may contribute to the probiotic properties of the *E. coli* strain (Guttsches et al., 2012). Another example is the *E. coli* K-12 strain, which shows a defective O-antigen structure, can be converted into a pathogen of *Caenorhabditis elegans* upon restoration of its O antigen structure (Browning et al., 2013). Further studies are ongoing to study how engineered LPS with different structures and components can be exploited to generate a spectrum of immuno-stimulatory molecules for the development of new adjuvants of vaccines and therapeutics (Needham et al., 2013).

## Future Perspectives

Microbiome derived-LPS play important roles in immune modulation and development of inflammatory diseases (Lin et al., 2016). LPS comprise of chemical molecules of very complicated components, though their basic constructions are similar. In concordance, LPS prepared from different bacterial species and strains produce gradient immune-modulation effects on host cells. Intriguingly, dualism that represents two abstract and complementary regulatory effect in LPS seems to exist, after identification and characterization of A-LPS. As counteracting activity exists between A- and P-LPS, engineering LPS structure and composition, or deploying relative LPS ratios may be used to either enhance or inhibit their activities on immunity and inflammation. Therefore, bacterial original or synthesized LPS may be used to modulate the immune response as a preventive or therapeutic measure for the management of chronic inflammation related diseases. Some difficulties and challenges are expected, especially on how to obtain optimal LPS species or determine relative compositions of A- and P-LPS for potential clinical applications. To circumvent the difficulties, use of modern techniques is proposed. A high-throughput experimentation workflow platform for rapidly and efficiently measuring cytokines, together with high efficacy chemical synthesis pipelines should be established to accelerate the optimization from laboratory-scale discovery to large-scale screening. In parallel, complementary artificial intelligence approaches are just coming into focus.

## Author Contributions

H-CL, T-LL: Conceptualization, original draft preparation. C-CL: Supervision and contents curation. C-CS: Messages provision and investigation. W-FL, Y-MC, J-JL, T-SW, C-MT: Validation, writing, and reviewing.

## Funding

We would like to express our thankfulness for funding provided from CORPD1F0013 and CORPD1J0051 from Chang Gung Memorial Hospital, 106-2320-B-182-028-MY3, 108-2321-B-182-002, 108-2320-B-030-005 from Ministry of Science and Technology (MOST), Microbiota Research Center from Chang Gung University, and the Research Center for Emerging Viral Infections from The Featured Areas Research Center Program within the framework of the Higher Education Sprout Project by the Ministry of Education (MOE) in Taiwan and MOST, Taiwan (MOST 108-3017-F-182 -001).

## Conflict of Interest

The authors declare that the research was conducted in the absence of any commercial or financial relationships that could be construed as a potential conflict of interest.
